# Enucleation of the Tonsil

**Published:** 1910-08-06

**Authors:** 


### ENUCLEATION OF THE TONSIL.

Every medical man who has had dealings with the tonsil will be familiar with two classes of case which give rise to difficulty and disappointment.

In the first there are the large tonsils which, after removal by the guillotine, again reach their original size in the space of a few months, and secondly, there are cases where, although the tonsil is so small that removal by the guillotine is practically impossible, yet constant attacks of tonsilitis and sore throats render the patient's life a misery.

In order fully to realise the meaning of these latter cases it is necessary to recall certain facts in connection with the tonsil. We all know it is a mass of lymphoid tissue in the angle between the pillars of the fauces; but how few of us fully realise that it is a mass of deep holes or crypts, in which myriads of organisms luxuriate, and, further, that the crypts are not mere superficial depressions, but long tunnels burrowing deeply into the substance of the organ.

In a well-cocainised throat these crypts may be explored with an ordinary seeker, and their number and extent will be a matter for surprise. In many instances they will be found to extend the whole thickness of the tonsil. It becomes obvious, therefore, that size alone is no criterion of the amount of trouble that a tonsil may cause; it is the crypts and their bacterial contents which are all-important. Some very large tonsils possess crypts which are small, shallow, and unimportant, and the trouble they occasion is only of a mechanical nature, owing to the obstruction they offer to respiration.

The worst tonsil of all is the small soft ragged tonsil with septic crypts, which is "buried" between the pillars of the fauces.

When it is remembered how deeply this kind of tonsil is embedded in the tonsillar fossa, it becomes very easy to understand how impossible it is, with any kind of guillotine, to remove the more deeply situated portion of the organ, in which the lower parts of the septic crypts are lying.

It is for cases such as these, as well as for cases of simple large tonsils which persist in growing after the use of the guillotine, that the operation of enucleation is now being frequently performed.

The advantage of completely ridding the patient of this troublesome and septic focus is obvious; it is necessary, however, to refer to the question which will naturally arise as to how patients get on with no tonsillar tissue at all, and, secondly, as to the difficulty and danger of the operation. The reply to the first question is that those people who appear to possess little or no tonsillar tissue naturally seem to be at no disadvantage; and secondly, the operation of enucleation has been performed many hundreds of times without causing any ill-effect *qua* the absence of tonsillar tissue.

As to the technique of the operation: it may be very easy, but in cases where there has been much chronic inflammation it is sometimes very difficult. Formerly carried out by careful dissection with the knife, it has now been found that the tonsil possesses a very distinct capsule which permits of its enucleation from the tonsillar bed by means of blunt instruments. The only real difficulties in the procedure are the maintenance of anæsthesia, and "hitting off" the capsule of the tonsil.

The anæsthetic should be chloroform, administered to the full surgical degree to avoid straining and coughing. With the patient on his back the tongue is then pulled forward and held in that position by a clip or fine thread passed through its substance. The tonsils then come prominently into view, and the next step is to seize one in a pair of forceps of a kind that will give a good grip without tearing away. And now comes the crux of the operation. It consists in gently scratching through the anterior pillar of the fauces one-eighth of an inch behind its free border. This should be done slowly and carefully with a fine-pointed pair of forceps (closed). When this is accomplished the greyish white capsule of the tonsil comes into view and the rest of the operation is easy.

Careful dissection with the forceps aided by the fingers will free the tonsil from its bed. It can then be turned inwards towards the middle line, when it will be found that below and posteriorly there is a small area of more close attachment where the vessels and lymphatics enter. A pressure forceps may be put on this pedicle, keeping close to the tonsil, which is then cut away with scissors.

The gap left after the removal of the tonsil will appear astonishingly large and deep, but will in the course of a few days rapidly close up and disappear.

Hæmorrhage may occasionally give trouble, but can be controlled by sponge pressure. In the majority of these operations hæmorrhage is much less than when the guillotine is used.

So satisfactory are the results that it is probable that enucleation will soon become the recognised method of dealing with troublesome tonsils.

